# Safety and Immunogenicity of a Sabin Strain-Based Inactivated Polio Vaccine: A Phase III, Randomized, Blinded, Positive-Control Clinical Trial in Infants Aged Two Months

**DOI:** 10.3390/vaccines14040312

**Published:** 2026-03-30

**Authors:** Hao Zhang, Yanjun Chen, Bidan Xu, Rong Tang, Yanting Wang, Jialei Hu, Shengqiu Yang, Mingwei Wei, Guifan Li, Qi Liang

**Affiliations:** 1 Beijing Minhai Biotechnology Co., Ltd., Beijing 102600, China; zhanghao@biominhai.com (H.Z.); xubidan@biominhai.com (B.X.); wangyanting@biominhai.com (Y.W.); yangshengqiu@biominhai.com (S.Y.); 2 Jiangsu Provincial Center for Disease Control and Prevention, Nanjing 210009, China; always_cyj@163.com (Y.C.); tangrongtr@126.com (R.T.); huctj@126.com (J.H.); js_wmw@163.com (M.W.)

**Keywords:** safety, immunogenicity, sIPV, primary immunization, booster immunization

## Abstract

**Objectives**: This randomized, blinded, positive-controlled phase III clinical trial aims to evaluate the safety and immunogenicity of the Sabin strain-based inactivated polio vaccine (sIPV) produced by Biominhai in healthy infants after primary and booster immunization. **Methods**: A total of 1200 healthy infants, aged 2 months, were randomly assigned to two groups in a 1:1 ratio to receive either one dose of sIPV or the control wIPV at 2, 3, and 4 months of age, followed by a booster dose at 18 months. The safety and immunogenicity of both the primary and the booster immunization were assessed. **Results**: The incidence of adverse reactions (AEs) was significantly lower in the sIPV group compared to the wIPV group after the primary immunization. Specifically, redness was the most frequently reported AE, occurring in 9% of the sIPV group versus 14% in wIPV (*p* = 0.01). Diarrhea was also less common in the sIPV group (3%) compared to the wIPV group (8%, *p* = 0.0004). Moreover, there were no significant differences in incidence, severity, or symptoms of AEs between the groups after the booster immunization. Most AEs were classified as grade 1, and notably, no serious AEs (SAEs) were associated with the trial vaccine. Seroconversion rates for types 1, 2, and 3 poliovirus neutralizing antibodies, in the sIPV group, exceeded 98% at 30 days after primary immunization and remained above 90% at 30 days after booster immunization. Notably, seroconversion rates for all three serotypes following both primary and booster immunizations were non-inferior to those observed in the wIPV group. Additionally, the geometric mean titers (GMTs) of neutralizing antibodies against all types were significantly higher in the sIPV group. **Conclusions**: The sIPV produced by Biominhai demonstrated comparable safety and immunogenicity to the control vaccine after both primary and booster immunizations.

## 1. Introduction

Poliomyelitis is a highly contagious acute disease caused by one of the three serotypes of poliovirus (types 1, 2, or 3). The infection is transmitted through fecal-oral and oral-oral routes and can invade the nervous system, leading to paralysis [1]. Children under the age of 5 are the most susceptible population, and infected individuals excrete the poliovirus through feces and respiratory secretions, typically for 4 and 2 weeks, respectively [2]. Currently, there is no effective cure for poliomyelitis, but it can be prevented through vaccination. Since the World Health Assembly resolved to initiate the Global Polio Eradication Initiative (GPEI) in 1988, the circulation of wild polioviruses (WPV) has been significantly controlled. The number of wild polio cases has decreased by more than 99%, from approximately 350,000 cases in over 125 endemic countries to only two remaining endemic countries (Pakistan and Afghanistan) [3,4].

There are two widely used poliomyelitis vaccines worldwide: the oral polio vaccine (OPV) and the inactivated polio vaccine (IPV). While OPV is easy to administer and provides long-lasting protection, it carries potential risks. OPV is composed of live attenuated poliovirus derived from the Sabin strains, which means that vaccination with OPV resembles natural exposure to the virus [5]. As a result, there is a possibility of vaccine-associated paralytic poliomyelitis (VAPP) and vaccine-derived poliovirus (VDPV), which can pose challenges to public health and hinder efforts to eradicate poliovirus [6,7]. In response to these concerns, a synchronized switch was implemented in 2016 following the global certification of wild poliovirus type 2 (WPV2) elimination in 2015 [2]. This switch replaced trivalent OPV (tOPV) with bivalent OPV (bOPV), which includes only types 1 and 3. To address potential immunity gaps resulting from the withdrawal of the type 2 component, at least one dose of IPV was introduced alongside the bOPV [8,9,10]. IPV is produced from inactivated strains of all three poliovirus types, thereby eliminating the risks associated with VAPP and the transmission of VDPV. However, traditional production of wild-type IPV (wIPV) using the Salk strain entails higher biosafety requirements, significantly increasing production costs and making IPV less accessible in low- and middle-income countries. As a solution, the World Health Organization (WHO) recommends using the less virulent Sabin strain instead of the wild-type Salk strain for IPV production, aiming to enhance the availability and affordability of IPV in developing countries [8,11,12].

This Phase III, randomized, blinded, positive-control clinical trial was initiated in October 2020 in Jiangsu Province, China. The study aimed to report the safety and immunogenicity findings from the Phase III clinical trial of the Sabin-IPV (sIPV) developed by Biominhai (Beijing Minhai Biotechnology Co., Ltd., Beijing, China) in healthy infants aged 2 months following both primary and booster immunizations.

## 2. Materials and Methods

### 2.1. Study Design

This randomized, double-blinded, positive-controlled phase III clinical trial was conducted by Jiangsu Provincial Center for Disease Control and Prevention (JSCDC) in Jiangsu Province, China, from October 2020 to October 2022. The study aimed to evaluate the safety and immunogenicity of a sIPV in infants aged two months (60~89 days, i.e., from the completion of 2 months to the day before reaching 3 months). A total of 1200 eligible participants were enrolled and randomly assigned to either the test or the control group on a 1:1 ratio. Participants in the test group received the trial vaccine sIPV developed by Biominhai (Beijing Minhai Biotechnology Co., Ltd.), while participants in the control group received a wIPV developed by Sanofi Pasteur. Both vaccines were administered at 2, 3, and 4 months of age for primary immunization, followed by a booster dose at 18 months of age. The vaccine was administered via intramuscular injection. The injection site was the anterolateral thigh for participants aged 2 to 11 months and the deltoid muscle of the upper arm for those aged 1 year and above. The trial was reviewed and approved by the Ethics Committee of JSCDC (approval No.: JSJK2017-A006-02) and conducted in accordance with the Declaration of Helsinki and Good Clinical Practice guidelines. Written informed consent was obtained from parents or legal guardians of all participants prior to enrollment. This clinical trial was registered at ClinicalTrials.gov (NCT07297186).

### 2.2. Participants

Eligible participants were infants aged 60 to 89 days, in good health and residing permanently within the study area, with no prior history of polio vaccination and no contraindications to vaccination, with written informed consent provided by the infant’s legal guardians who demonstrated willingness and ability to comply with all requirements of the clinical trial protocol and had an axillary temperature ≤ 37.0 °C at the time of enrollment. The inclusion criteria: (1) Healthy permanent residents aged 2 months (60~89 days); (2) Infant’s legal guardians agree to sign the informed consent forms; (3) Infant’s legal guardians are able to comply with the requirements of the clinical trial protocol; (4) Axillary temperature ≤ 37.0 °C. The exclusion criteria: (1) Preterm birth (gestational age < 37 weeks); (2) Presence of congenital malformations, developmental disorders, genetic defects, or severe malnutrition; (3) History of poliomyelitis; (4) Personal or family history of allergy, convulsions, epilepsy, encephalopathy, or psychiatric disorders; (5) Known hypersensitivity to any component of the study vaccine or a history of severe allergic reaction (e.g., anaphylaxis) to any previous vaccination; (6) Immunodeficiency or receipt of immunosuppressive therapy; (7) Diagnosed coagulation disorders (including factor deficiencies, coagulopathies, and platelet abnormalities) or evidence of significant bruising/bleeding diathesis; (8) Known or investigator-suspected concurrent acute, active chronic diseases (including respiratory, cardiovascular, hepatic, renal, or dermatological conditions), acute infection, or maternal HIV infection; (9) Occurrence of fever (axillary temperature ≥ 38.0 °C) within the 3 days preceding enrollment; (10) Acute illness requiring systemic antibiotic or antiviral treatment within the 7 days preceding enrollment; (11) Administration of blood products within the 3 months preceding enrollment; (12) Receipt of any live attenuated vaccine within the 14 days preceding enrollment; (13) Receipt of any inactivated or subunit vaccine within the 7 days preceding enrollment; (14) Recent administration of any other investigational product and any other condition deemed by the investigator as potentially interfering with the assessment of trial outcomes.

### 2.3. Vaccine

The trial vaccine sIPVs were developed by Biominhai (Beijing Minhai Biotechnology Co., Ltd., Beijing, China), containing 15, 45, and 45 D-antigen units (DU) for types 1, 2, and 3 polioviruses, respectively. The control vaccine wIPVs were manufactured by Sanofi Pasteur, with 40 DU for type 1, 8 DU for type 2, and 32 DU for type 3. The control vaccine (Imovax Polio^®^, Sanofi Pasteur, Lyon, France) was administered with lot number R3N141M for the primary immunization and lot number U3E331M for the booster immunization. Both vaccines were sterile liquid formulations (0.5 mL/dose) intended for intramuscular injection.

### 2.4. Randomization and Masking

Vaccine randomization and blinding were performed using SAS software Version 9.4 (SAS Institute Inc., Cary, NC, USA) with predetermined block lengths according to the randomization code. Eligible participants were sequentially assigned vaccine identification numbers in ascending order based on their chronological order of enrollment. The trial and control vaccines were packaged in identical external containers but with distinct internal packaging. The investigator responsible for vaccine preparation and administration was unblinded to subject allocation but was strictly prohibited from disclosing this information to any other personnel, indicating that participants, investigators (including outcome assessors and safety assessors), statisticians, and the sponsor did not participate in any other research activities. All other investigators, as well as participants, remained blinded to treatment assignments.

### 2.5. Safety Assessment

All solicited and unsolicited adverse events (AEs) were recorded. AEs assessed by the investigator as causally related to the study vaccine were defined as adverse drug reactions. Solicited local symptoms included tenderness, induration, swelling, rash, redness, and cellulitis. Solicited systemic symptoms comprised fever, diarrhea, anorexia, vomiting, new-onset convulsions, cough, mucocutaneous abnormality, irritability or lethargy, and acute allergic reactions. The severity of AEs was graded according to the clinical trial guidelines of preventive vaccines issued by the China Medical Products Administration [13]. All the participants were monitored for AEs at the vaccination site for 30 min after each vaccination. For the subsequent 30 days post-vaccination, parents/guardians completed safety observations using diary cards, verified during scheduled visits where investigators investigated them. A serious adverse event (SAE) is any adverse medical event that, following administration of the investigational product, results in death, is life-threatening, causes persistent or significant disability or incapacity, requires inpatient hospitalization or prolongation of existing hospitalization, or is a congenital anomaly or birth defect. Serious adverse events (SAEs) occurring from the first vaccination through 12 months post-booster were monitored via caregiver reporting and investigator follow-up (either on telephone or in-person). All reported SAEs were investigated and verified by the investigators.

### 2.6. Immunogenicity Assessment

Venous blood samples (approximately 2 mL) were collected before the first vaccination, 30 days after completion of the primary series, prior to the booster dose, and 30 days after the booster. Serum samples were tested for neutralizing antibody levels using the micro-neutralization assay conducted by the National Institute for Food and Drug Control, China (NIFDC). The challenge viruses were Sabin strains: types 1, 2, and 3. Hep-2 cells (human laryngeal carcinoma epithelial cells) were used as the cell line. Serum samples were serially diluted two-fold starting at 1:4. Each dilution was mixed with the corresponding challenge virus and incubated at 35 °C for 3 h, followed by the addition of Hep-2 cell suspension. Following virus inoculation, cell growth was observed under a microscope on day 2, cytopathic effects (CPE) were assessed on day 4, and final results were determined on day 7. Neutralizing antibody titers were calculated using the Karber method, defined as the highest serum dilution that protected 50% of cells against infection with 100 CCID_50_ of the challenge virus. Each assay run included a reference serum with a known neutralizing antibody titer to ensure consistency and reproducibility. The primary endpoint was the seroconversion rates of neutralizing antibodies 30 days after the primary immunization. The secondary endpoints were the geometric mean titers (GMTs), the fold increase in GMT, and the seropositivity rate of antibodies 30 days after the primary and booster immunization. Seropositive was defined as neutralizing antibody titers ≥ 1:8 against poliovirus types 1, 2, and 3. Seroconversion was defined as achieving a protective antibody level post-vaccination in participants without a protective pre-vaccination level, or as a ≥4-fold increase in antibody levels post-vaccination in participants with a protective pre-vaccination baseline.

### 2.7. Statistical Analysis

All statistical analyses were performed using SAS Version 9.4. The incidence of AEs between the test and control groups was compared using the Chi-square test or Fisher’s exact test. The differences in seroconversion rates and seropositive rates between the two groups were assessed using the Chi-square test, with the two-sided 95% confidence intervals (CIs) calculated. Non-inferiority of seroconversion rates was defined as the lower bound of the 95% CI for the difference between groups exceeding the predefined inferiority margin of −10%. The differences in GMTs and the fold increase in GMTs between groups were compared using the independent two-sample *t*-test or *t*’-test, with 95% CI also calculated. For baseline characteristics of the subjects, comparisons of age and BMI between groups were performed using the *t*-test, while gender differences were analyzed using the Chi-square test, and ethnicity differences were assessed with Fisher’s exact test. *p*-value ≤ 0.05 was considered statistically significant.

## 3. Results

### 3.1. Study Participants

**Figure 1:**
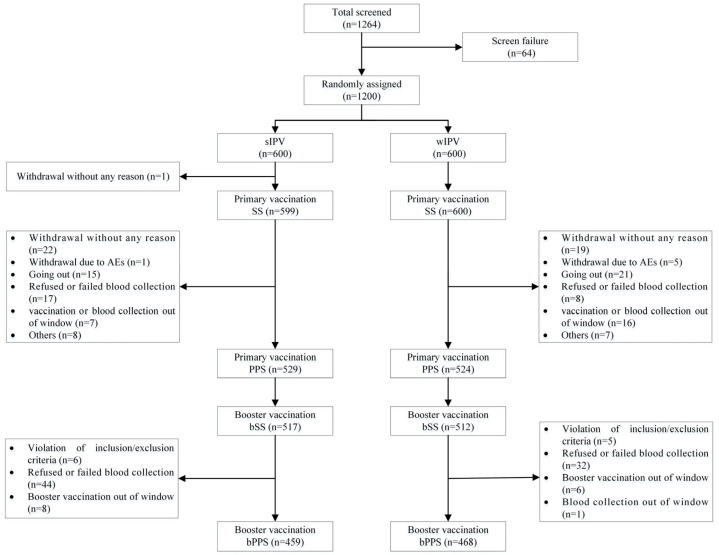
Participant flow through the study. SS, safety analysis set; PPS, per-protocol analysis set for primary immunogenicity analysis. bSS, safety analysis set for booster immunization; bPPS, per-protocol analysis set for booster immunogenicity analysis.

At the primary immunization stage, a total of 1200 healthy participants were enrolled, with 600 in each group. Among all participants, 1199 (599 in the sIPV group and 600 in the wIPV group) received at least one dose of the vaccine and underwent at least one safety observation, making them eligible for the safety analysis set (SS). 1053 participants (529 in the sIPV group and 524 in the wIPV group) completed three doses of the primary immunization, pre- and post-immunization blood collection, and had valid antibody results, qualifying for the per-protocol analysis set (PPS). In the booster immunization stage, 1029 participants (517 in the sIPV group and 512 in the wIPV group) completed the booster immunization and were included in the booster immunization SS (bSS). Among these, 927 participants (459 in the sIPV group and 468 in the wIPV group) completed pre- and post-booster blood collection and had valid antibody results, qualifying them for the booster immunization PPS (bPPS). The main reasons for exclusion from PPS/bPPS were withdrawal without any specific reason, refusal or failure of blood collection, participants going out, and vaccination or blood collection out of the window. The flowchart of the participants is shown in Figure 1.

At enrollment, the mean age was 2.40 months for the sIPV group and 2.39 months for the wIPV group. Detailed baseline characteristics are provided in Table 1. No significant differences were observed between the two groups in terms of age, sex, race, axillary temperature, BMI, and the proportion of susceptible subjects for types 2 and 3 prior to both primary and booster immunization. Before primary immunization, the proportion of susceptible subjects for type 1 was higher in the sIPV group than in the wIPV (*p* = 0.02), while no differences were observed prior to booster immunization. The GMTs for all three poliovirus types did not differ significantly between the two groups before primary immunization. Prior to the booster immunization, the GMTs for types 1, 2, and 3 in the sIPV were significantly higher than those in the wIPV group (all *p* < 0.0001).

### 3.2. Safety

**Figure 2:**
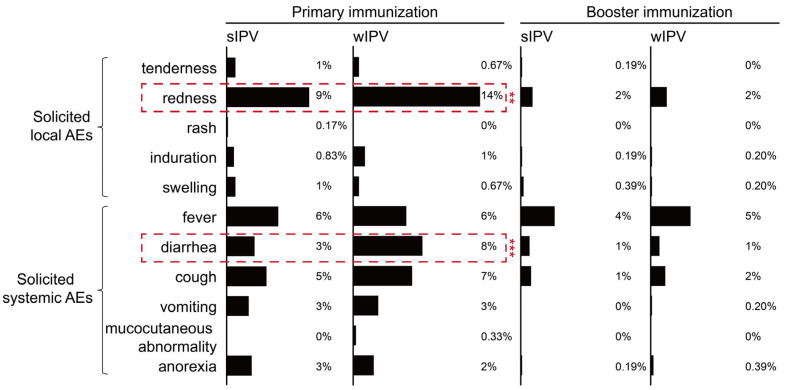
Solicited local AEs or solicited systemic AEs within 30 days after primary and booster immunization. **, *p* < 0.01. ***, *p* < 0.001.

Table 2 illustrates the incidence of overall adverse drug reactions in both groups. From the first dose to 30 days after primary immunization, the sIPV group had a significantly lower incidence of total AEs (24%) compared to the wIPV group (33%) (*p* = 0.0009). Specifically, 11% of participants in the sIPV group experienced solicited local AEs, lower than the 15% in the wIPV group (*p* = 0.02). Redness was the most common solicited local AE, occurring in 9% of the sIPV, compared to 14% in the wIPV group (*p* = 0.01) (Figure 2). Furthermore, 14% of participants in the sIPV reported solicited systemic AEs, compared to 21% in the wIPV (*p* = 0.0041). Fever, cough, and diarrhea were the primary symptoms, with diarrhea occurring significantly less frequently in the sIPV (3%) compared to the wIPV (8%, *p* = 0.0004) (Figure 2). No significant differences were observed between groups for other solicited symptoms. The incidence of unsolicited AEs was 3% in both groups, with symptoms, including retching and crying. Importantly, most AEs were classified as Grade 1 in severity, and the incidence of total AEs, solicited local and systemic AEs was consistently lower in the sIPV group than in the wIPV.

Following booster immunization, the incidence of total AEs within 30 days was 8% in the sIPV and 10% in the wIPV. In the sIPV group, 2% of participants experienced solicited local AEs, primarily redness (2%). Solicited systemic AEs were reported by 6% of participants, with fever (4%) being the most common, followed by diarrhea, cough, and anorexia (Figure 2). In the wIPV group, the incidence of solicited local AEs was 3%, and solicited systemic AEs was 8%. Moreover, the majority of reported AEs in both groups were classified as Grade 1 in severity. There were no significant differences between groups in terms of the incidence, severity, and symptoms of AEs, and neither group reported any unsolicited AEs.

The incidence of AEs following each injection is shown in Table 3. AEs primarily occurred after the first dose of immunization, with an incidence of 12% in the sIPV group, lower than the 17% observed in the wIPV group (*p* = 0.02). After the second dose, the incidence was 9% and 11% in the sIPV and wIPV, respectively. Following the third dose, the incidence of AEs significantly differed in the sIPV (9%) compared with the wIPV (14%) (*p* = 0.01). After the booster dose, the incidence of AEs was 8% in the sIPV and 10% in the wIPV. Within 12 months after the booster dose, the incidence of serious adverse events (SAEs) was lower in the sIPV group (4%, 26 participants) compared to the wIPV group (7%, 44 participants) (*p* = 0.03). Importantly, all participants who experienced SAEs had fully recovered and were discharged. According to the investigators, none of the SAEs was related to vaccination.

### 3.3. Primary Immunogenicity

Thirty days after primary vaccination, seroconversion rates for type 1 poliovirus neutralizing antibodies were 99% in the test group and 96% in the control group; for type 2, 98% vs. 87%, with significantly higher responses observed in the test group for both serotypes (type 1, *p* = 0.01; type 2, *p* < 0.0001); seroconversion for type 3 was identical between groups (99%). Non-inferiority was confirmed for all three serotypes. The GMTs for type 1 were 2924.68 in the test group and 380.41 in the control group; for type 2 were 957.64 and 242.85; and for type 3 were 1516.95 and 779.86, respectively. The test group demonstrated significantly higher GMTs for all three serotypes (all *p* < 0.0001). The fold increase in GMT was also significantly higher in the test group for all three serotypes (314.90 vs. 37.25 for type 1; 106.14 vs. 25.51 for type 2; and 252.22 vs. 130.01 for type 3, all *p* < 0.0001). Seropositivity rates reached 100% for all three serotypes in both groups (Table 4).

### 3.4. Booster Immunogenicity

At 30 days post-booster vaccination, lower seroconversion rates were observed in the test group for type 1 (90% vs. 95%, *p* = 0.0043) and type 3 (95% vs. 99%, *p* = 0.01), whereas responses to type 2 were comparable between the two groups (95% vs. 94%). The GMTs for type 1 were 10,446.55 in the test group and 4621.78 in the control group; for type 2, 11,280.62 vs. 3889.81; and for type 3, 8278.72 vs. 7148.98. The test group demonstrated significantly higher GMTs for all serotypes (*p* < 0.0001, *p* < 0.0001, and *p* = 0.01, respectively). The fold increases in GMTs were 15.11 and 23.52 for poliovirus type 1, and 30.21 and 62.03 for type 3 in the test and control groups, respectively, with significantly lower increases in the test group for both serotypes (both *p* < 0.0001). For type 2, the fold increase did not differ significantly between the two groups (24.27 vs. 25.47). Seropositivity rates reached 100% for all three poliovirus types in both groups (Table 5).

## 4. Discussion

OPV can lead to VAPP and VDPV in rare cases, but the introduction of IPV has significantly reduced these risks [14]. The current immunization schedule for poliovirus vaccines in China consists of two doses of IPV followed by two doses of bOPV. Based on the superior safety and affordability of the sIPV, Biominhai developed the sIPV to meet market demand. In the Phase II clinical trials of the sIPV, immunizations were conducted using low-, medium-, and high-D antigen content, all of which exhibited good safety profiles and immunogenicity [15]. Furthermore, a sequential immunization regimen employing “2 sIPV + 1 bOPV” demonstrated that the sIPV was non-inferior to the control vaccine in terms of safety and immunogenicity [16]. This study primarily reports the results from the Phase III clinical trial of sIPV.

In the study, no SAEs related to the trial vaccine sIPV were reported, indicating good tolerability. AEs predominantly occurred after the first dose, with the majority classified as grade 1 (mild) in severity, and no grade 3 or higher AEs were observed. The most frequently observed solicited local AE in this study was redness, whereas fever was the most common solicited systemic AE. These findings are consistent with observations from the phase II clinical trial and sequential immunization studies of the test vaccine sIPV, as well as from other published studies [15,16,17,18]. Following primary immunization, the overall incidence of AEs associated with the sIPV group was lower than that of the wIPV. After booster immunization, no significant differences were observed between groups regarding the incidence, severity, and symptoms of AEs. Overall, the test vaccine sIPV demonstrated a favorable safety profile.

The trial vaccine elicited a robust immune response following both the three-dose primary immunization series and the booster dose. Thirty days after primary immunization, higher seroconversion rates were observed in the sIPV group for type 1 (99% vs. 96%, *p* = 0.01) and type 2 (98% vs. 87%, *p* < 0.0001); type 3 was identical between groups (99%). Following booster immunization at 30 days, the seroconversion rates for type 1 (90% vs. 95%, *p* = 0.0043) and type 3 (95% vs. 99%, *p* = 0.01) were lower in the sIPV group, whereas those for type 2 were essentially equivalent between the two groups (95% vs. 94%). The significantly higher neutralizing antibody levels observed at the pre-booster immune baseline in the sIPV group may have contributed to lower seroconversion rates for types 1 and 3 compared with the control group. It is important to note that, after both primary and booster immunizations, the seroconversion rates for all types in the sIPV group were non-inferior to those in the control group, and the GMTs for all three types were significantly higher in the sIPV group. The D-antigen level for type 1 in the sIPV (15 DU) is lower than that in the wIPV (40 DU); nevertheless, the GMT remains significantly higher in the sIPV group compared to the wIPV group, both after primary immunization (2924.68 vs. 380.41) and after booster immunization (10,446.55 vs. 4621.78). The D-antigen level for type 2 in the wIPV (8 DU) is lower than that in the sIPV (45 DU), which may contribute to the observed differences in GMTs for type 2 during both primary immunization (957.64 vs. 242.85) and booster immunization (11,280.62 vs. 3889.81).

When compared with other Sabin-IPV preparations that have been previously evaluated, the seropositivity and seroconversion rates observed in this study are generally comparable. In a phase III trial conducted in China using a different Sabin-IPV product, seroconversion rates after primary immunization were reported as 100% (type 1), 94.9% (type 2), and 99.0% (type 3), respectively [19]. Similarly, another study evaluating a Sabin-IPV produced by a different manufacturer reported seroconversion rates between 95.8% and 99.2% following primary immunization [20]. These findings are consistent with the results of the present study, in which seroconversion rates for the sIPV group were 99.7% (type 1), 97.60% (type 2), and 100.00% (type 3), with seropositivity rates of 100% for all three serotypes [21], which aligns with the results observed in the control group of this study.

The excellent safety and immunogenicity results led to the approval of the sIPV by the China National Medical Products Administration for market release. The sIPV developed by Biominhai shows potential as a candidate vaccine for future immunization strategies anticipated by the WHO, which may shift from the sequential use of bOPV and IPV to exclusive IPV administration [2]. Nevertheless, this study has certain limitations. First, participants did not include preterm infants or individuals with immunocompromised conditions, which may affect the generalizability of the results. Second, immunogenicity was evaluated only at approximately 30 days after the final vaccination; therefore, further studies are warranted to assess longer-term antibody persistence. In addition, IPV is commonly administered concomitantly with other vaccines. Previous studies suggest that co-administration of sIPV with routine childhood vaccines, including diphtheria-tetanus-acellular pertussis vaccine, hepatitis A vaccine, and measles-mumps-rubella vaccine, does not compromise safety or immunogenicity [22,23,24]. If necessary, future studies will further assess the safety and immunogenicity of concomitant administration of sIPV with additional vaccines, such as the hib vaccine, and will evaluate lot-to-lot consistency.

## 5. Conclusions

The trial vaccine, sIPV developed by Biominhai, exhibited superior safety compared to the control vaccine following primary immunization, while no statistically significant difference was observed after the booster dose. Furthermore, the sIPV elicited a robust immune response, exhibiting non-inferior immunogenicity compared to the control vaccine after both primary and booster immunizations. In summary, sIPV manufactured by Biominhai demonstrated comparable safety and immunogenicity to the control vaccine after both primary and booster immunizations in infants aged 2 months.

## Figures and Tables

**Table 1:** Baseline characteristics.

	Primary Immunization			Booster Immunization		
	sIPV (N = 529)	wIPV (N = 524)	*p* Value	sIPV (N = 517)	wIPV (N = 512)	*p* Value
Age (months, Mean ± SD)	2.40 ± 0.26	2.39 ± 0.27	0.54	2.39 ± 0.26	2.38 ± 0.27	0.59
Sex						
Female, n (%)	264 (50)	261 (50)	0.98	241 (52)	235 (50)	0.51
Male, n (%)	265 (50)	263 (50)		224 (48)	238 (50)	
Race						
Han, n (%)	525 (99)	521(99)	1.00	461 (99)	469 (99)	1.00
Other, n(%)	4 (1)	3 (1)		4 (1)	4 (1)	
Axillary temperature (°C, Mean ± SD)	36.57 ± 0.29	36.58 ± 0.31	0.61	36.56 ± 0.29	36.58 ± 0.30	0.51
BMI (kg/m^2^, Mean ± SD)	17.56 ± 1.85	17.65 ± 1.90	0.43	17.55 ± 1.87	17.72 ± 1.92	0.18
Type 1 poliovirus						
Pre-immune susceptible subjects, n (%)	269 (51)	228 (44)	0.02	1 (0.22%)	2 (0.43%)	1.00
Pre-immune GMT ± GSD (95% CI)	9.29 ± 2.75 (8.52~10.12)	10.21 ± 2.69 (9.38~11.12)	0.12	691.58 ± 3.30 (619.78~771.69)	196.47 ± 2.84 (178.68~216.04)	<0.0001
Type 2 poliovirus						
Pre-immune susceptible subjects, n (%)	262 (50)	256 (49)	0.83	0 (0)	4 (1)	0.12
Pre-immune GMT ± GSD (95% CI)	9.02 ± 2.68 (8.29~9.81)	9.52 ± 2.78 (8.72~10.39)	0.39	464.85 ± 2.84 (422.34~511.64)	152.70 ± 3.18 (137.47~169.62)	<0.0001
Type 3 poliovirus						
Pre-immune susceptible subjects, n (%)	391 (74)	381 (73)	0.66	7 (2)	13 (3)	0.19
Pre-immune GMT ± GSD (95% CI)	6.01 ± 2.23 (5.62~6.44)	6.00 ± 2.19 (5.61~6.42)	0.96	274.02 ± 3.45 (244.57~307.02)	115.24 ± 3.59 (102.60~129.44)	<0.0001

Booster pre-immune susceptible subjects and GMT were analyzed in the bPPS.

**Table 2:** Incidence of overall AEs within 30 days after primary and booster immunization.

	Primary Immunization			Booster Immunization		
	sIPV (N = 599)	wIPV (N = 600)	*p* Value	sIPV (N = 517)	wIPV (N = 512)	*p* Value
Total AEs	143 (24%)	195 (33%)	0.0009	40 (8%)	52 (10%)	0.17
Grade 1	118 (20%)	166 (28%)	0.0012	20 (4%)	25 (5%)	0.43
Grade 2	41 (7%)	52 (9%)	0.24	22 (4%)	29 (6%)	0.30
Grade 3	0	0	/	1 (0.19%)	2 (0.39%)	0.62
Solicited local AEs	64 (11%)	92 (15%)	0.02	8 (2%)	13 (3%)	0.26
Grade 1	53 (9%)	82 (14%)	0.01	7 (1%)	8 (2%)	0.78
Grade 2	15 (3%)	13 (2%)	0.70	1 (0.19%)	5 (1%)	0.12
Solicited systemic AEs	86 (14%)	124 (21%)	0.0041	33 (6%)	41 (8%)	0.31
Grade 1	67 (11%)	97 (16%)	0.01	14 (3%)	17 (3%)	0.57
Grade 2	28 (5%)	40 (7%)	0.14	21 (4%)	25 (5%)	0.52
Grade 3	0	0	/	1 (0.19%)	2 (0.39%)	0.62
Unsolicited AEs	17 (3%)	15 (3%)	0.72	0	0	/
Grade 1	16 (3%)	15 (3%)	0.85	0	0	/
Grade 2	1 (0.17%)	0	0.50	0	0	/

Grade 1 is mild; Grade 2 is moderate; Grade 3 is severe.

**Table 3:** Incidence of AEs of each injection.

	sIPV	wIPV	*p* Value
Primary first injection, n/N_SS1_	73/599 (12%)	102/600 (17%)	0.02
Primary second injection, n/N_SS2_	51/583 (9%)	66/578 (11%)	0.13
Primary third injection, n/N_SS3_	50/568 (9%)	76/562 (14%)	0.01
Booster injection, n/N_bSS_	40/517 (8%)	52/512 (10%)	0.17

N_SS1_/N_SS2_/N_SS3_/N_bSS_, the number of infants who received the primary 1st/2nd/3rd/booster injection.

**Table 4:** Immunogenicity analysis in PPS within 30 days after primary immunization.

Primary Immunization	sIPV (N = 529)	wIPV (N = 524)	*p* Value	Difference% (95% CI)	Non-Inferiority * Yes/No
Type 1 poliovirus					
Seropositive (%) (95% CI)	100 (99.31~100.00)	100 (99.30~100.00)	/		
Seroconversion (%) (95% CI)	99 (97.29~99.47)	96 (93.94~97.50)	0.01	2.68 (0.74~4.63)	Yes
GMT ± GSD (95% CI)	2924.68 ± 2.84 (2675.38~3197.21)	380.41 ± 2.13 (356.44~405.99)	<0.0001		
Fold increase in GMT (95% CI)	314.90 ± 5.33 (272.97~363.27)	37.25 ± 3.62 (33.36~41.60)	<0.0001		
Type 2 poliovirus					
Seropositive (%) (95% CI)	100 (99.31~100.00)	100 (99.30~100.00)	/		
Seroconversion (%) (95% CI)	98 (96.31~98.96)	87 (84.05~89.95)	<0.0001	10.71 (7.60~13.81)	Yes
GMT ± GSD (95% CI)	957.64 ± 2.32 (891.11~1029.14)	242.85 ± 2.44 (224.96~262.17)	<0.0001		
Fold increase in GMT (95% CI)	106.14 ± 4.45 (93.43~120.58)	25.51 ± 4.90 (22.26~29.24)	<0.0001		
Type 3 poliovirus					
Seropositive (%) (95% CI)	100 (99.31~100.00)	100 (99.30~100.00)	/		
Seroconversion (%) (95% CI)	99 (97.81~99.69)	99 (97.27~99.46)	0.55	0.39 (−0.89~1.67)	Yes
GMT ± GSD (95% CI)	1516.95 ± 2.38 (1408.62~1633.61)	779.86 ± 2.52 (720.47~844.16)	<0.0001		
Fold increase in GMT ± GSD (95% CI)	252.22 ± 3.62 (225.98~281.51)	130.01 ± 3.62 (116.43~145.17)	<0.0001		

* Non-inferiority was achieved if the lower limit of the 95% CI of the difference sIPV–wIPV for the seroconversion was >−10%.

**Table 5:** Immunogenicity analysis in the bPPS within 30 days after booster immunization.

Booster Immunization	sIPV (N = 517)	wIPV (N = 512)	*p* Value	Difference% (95% CI)
Type 1 poliovirus				
Seropositive (%) (95% CI)	100 (99.20~100.00)	100 (99.21~100.00)	/	
Seroconversion (%) (95% CI)	90 (87.10~92.76)	95 (92.72~96.86)	0.0043	−4.89 (−8.24~−1.54)
GMT±GSD (95% CI)	10,446.55 ± 1.85 (9872.91~11,053.53)	4621.78 ± 2.20 (4302.68~4964.55)	<0.0001	
Fold increase in GMT ± GSD (95% CI)	15.11 ± 3.10 (13.62~16.76)	23.52 ± 3.37 (21.07~26.27)	<0.0001	
Type 2 poliovirus				
Seropositive (%) (95% CI)	100 (99.20~100.00)	100 (99.21~100.00)	/	
Seroconversion (%) (95% CI)	95 (93.09~97.15)	94 (91.97~96.34)	0.50	0.98 (−1.84~3.80)
GMT ± GSD (95% CI)	11,280.62 ± 1.78 (10,697.06~11,896.02)	3889.81 ± 2.32 (3603.87~4198.44)	<0.0001	
Fold increase in GMT ± GSD (95% CI)	24.27 ± 3.15 (21.84~26.96)	25.47 ± 3.67 (22.64~28.67)	0.55	
Type 3 poliovirus				
Seropositive (%) (95% CI)	100 (99.20~100.00)	100 (99.21~100.00)	/	
Seroconversion (%) (95% CI)	95 (93.09~97.15)	99 (96.94~99.40)	0.01	−3.08 (−5.28~−0.87)
GMT ± GSD (95% CI)	8278.72 ± 2.18 (7708.81~8890.76)	7148.98 ± 2.34 (6616.55~7724.25)	0.01	
Fold increase in GMT ± GSD (95% CI)	30.21 ± 3.46 (26.96~33.85)	62.03 ± 3.45 (55.43~69.43)	<0.0001	

## Data Availability

The original contributions presented in this study are included in the article. Further inquiries can be directed to the corresponding authors.
